# Terapia Hormonal e Hipertensão em Mulheres na Pós-Menopausa: Resultados do Estudo Longitudinal de Saúde do Adulto (ELSA-Brasil)

**DOI:** 10.36660/abc.20210218

**Published:** 2022-05-04

**Authors:** Luana Ferreira-Campos, Ligia Gabrielli, Maria da Conceição Chagas Almeida, Estela Maria Leão Aquino, Sheila Maria Alvim Matos, Rosane Harter Griep, Roque Aras

**Affiliations:** 1 Universidade Federal da Bahia Programa de Pós-graduação em Medicina e Saúde Salvador BA Brasil Universidade Federal da Bahia - Programa de Pós-graduação em Medicina e Saúde, Salvador, BA – Brasil; 2 Universidade Federal da Bahia Instituto de Saúde Coletiva Salvador BA Brasil Universidade Federal da Bahia - Instituto de Saúde Coletiva, Salvador, BA – Brasil; 3 Fundação Oswaldo Cruz Instituto Gonçalo Moniz Salvador BA Brasil Fundação Oswaldo Cruz (Fiocruz) - Instituto Gonçalo Moniz, Salvador, BA – Brasil; 4 Fundação Oswaldo Cruz Laboratório de Educação em Ambiente e Saúde Rio de Janeiro RJ Brasil Fundação Oswaldo Cruz (Fiocruz) - Laboratório de Educação em Ambiente e Saúde, Rio de Janeiro, RJ – Brasil; 5 Universidade Federal da Bahia Faculdade de Medicina da Bahia Salvador BA Brasil Universidade Federal da Bahia Faculdade de Medicina da Bahia, Salvador, BA – Brasil

**Keywords:** Hipertensão, Pós-Menopausa, Terapia de Reposição Hormonal

## Abstract

**Fundamento:**

A hipertensão arterial é considerada um importante fator de risco de morbidade e mortalidade cardiovascular em mulheres na pós-menopausa. Embora a terapia hormonal da menopausa (THM) seja um tratamento muito eficiente para sintomas vasomotores nesse período, a influência dessa terapia na pressão arterial ainda não está clara.

**Objetivo:**

Avaliar a relação entre o uso de THM e a hipertensão em participantes do ELSA-Brasil.

**Métodos:**

Um estudo transversal usando dados da linha de base da coorte ELSA-Brasil, com 2.138 mulheres que passaram por menopausa natural. Neste estudo, foi analisado a hipertensão, definida como pressão arterial ≥140/90 mmHg ou uso anterior de anti-hipertensivo, e o uso da THM, com participantes sendo classificadas em grupos daquelas que nunca usaram, que já usaram e que estavam em uso atual. As associações foram avaliadas usando-se um modelo de regressão logística multivariada com uma significância estatística definida em p<0,05.

**Resultados:**

No total, 1.492 mulheres (69,8%) nunca tinham usado a THM, 457 (21,4%) tinham usado no passado, e 189 (8,8%) estavam em uso atual. O uso de THM foi mais comum em mulheres que tinham índice de massa corporal <25 kg/m^2^ e níveis de triglicérides <150 mg/dl, que eram fisicamente menos inativas, não fumantes e não diabéticas. As mulheres em uso atual da THM apresentaram menores chances de ter hipertensão (OR=0,59; IC 95%: 0,41-0,85), em comparação com as que nunca a usaram. Na maioria dos casos, a THM foi iniciada com idade até 59 anos, com menos de 10 anos de menopausa e o uso durou até cinco anos.

**Conclusão:**

O uso atual da THM não esteve relacionado à hipertensão, especialmente em mulheres saudáveis e que tinham menos de 60 anos de idade.

## Introdução

A hipertensão arterial é um importante fator de risco para morbidade e mortalidade cardiovascular em mulheres na pós-menopausa.^1^ A transição menopausal é responsável por modificações que podem favorecer a hipertensão e outros fatores de risco cardiovascular. As alterações dos hormônios sexuais endógenos e a própria fisiologia do envelhecimento podem afetar a função cardíaca, rigidez arterial, resistência à insulina, perfil lipídico, aumento do peso corporal e a adiposidade central.^1 , 2^

Embora a terapia hormonal da menopausa (THM) seja o tratamento mais eficiente para sintomas vasomotores e para a síndrome genitourinária da menopausa, e muito efetivo para a prevenção de perda óssea e fraturas,^3^ outros efeitos também estão envolvidos e esse tratamento pode estar associado a marcadores de risco cardiovascular.^4 , 5^ Os riscos e benefícios do uso da THM parecem depender do tipo de hormônio prescrito, da dose e da duração do uso, da via de administração e do momento em que o tratamento foi iniciado.^3^

Achados relacionados ao efeito da THM na pressão arterial em mulheres têm sido conflitantes, com ensaios clínicos relatando ou um efeito neutro^6 , 7^ ou um efeito protetor com redução da pressão arterial,^8 , 9^ enquanto outros, com o mesmo desenho, sugerem efeitos negativos com um aumento nos níveis de pressão arterial.^10 , 11^

Considerando que a maioria dos estudos que lidam com esse assunto foram realizados com amostras de mulheres norte-americanas e europeias, é necessário avaliar o efeito da THM na pressão arterial de mulheres brasileiras. Partindo da hipótese de que a THM afeta os níveis de pressão arterial, este estudo teve o objetivo de avaliar a relação entre uso de THM e hipertensão em mulheres que participam do Estudo Longitudinal de Saúde do Adulto (ELSA-Brasil).

## Materiais e métodos

### Desenho e população do estudo

Este estudo analisou dados da linha de base do ELSA-Brasil (2008-2010), uma coorte multicêntrica composta por 15.105 funcionários públicos que trabalham na educação superior pública e em institutos de pesquisa em seis cidades brasileiras. Desses participantes, 8.218 eram mulheres. Detalhes sobre o estudo já foram publicados em outros lugares.^12^

Para a presente análise, 2.138 mulheres que tinham passado pela menopausa natural e eram normotensas, ou que haviam recebido um diagnóstico de hipertensão após a menopausa, foram avaliadas. Foram excluídas 1.453 participantes que referiram menopausa cirúrgica ou induzida por tratamento, histórico de falência ovariana prematura, que relataram uso da THM ou ter recebido um diagnóstico de hipertensão em período anterior à menopausa. Nas análises especificamente relacionadas ao tempo de uso ou da data de início do uso da THM, algumas participantes foram excluídas devido a dados não informados.

### Coleta de dados

Uma equipe treinada, certificada para realizar cada procedimento, realizou a coleta de dados. Foi implementado um sistema rigoroso de controle de qualidade.^13^ Foram realizadas entrevistas presenciais usando questionários padronizados, e testes clínicos e laboratoriais foram realizados nos centros de pesquisa.^12^

### Menopausa e THM

As participantes que responderam “não” à pergunta: “Você ainda menstrua?”, e que, além disso, relataram não menstruar “há mais de 1 ano” foram consideradas na menopausa.^14^ O tipo de menopausa foi investigado a partir da resposta da participante à pergunta: “Por que você não menstrua mais?”. A idade em que entrou na menopausa foi determinada a partir de respostas à pergunta: “Quantos anos você tinha quando você menstruou pela última vez?”.

Em relação ao uso de THM, as participantes foram questionadas: “Você usa ou já usou medicamentos contendo hormônios femininos para aliviar os sintomas da menopausa?” e “Você está usando medicamentos contendo hormônios femininos atualmente para aliviar os sintomas da menopausa?”. Essas duas perguntas foram combinadas para se obter a variável da exposição. O padrão da THM foi avaliado categoricamente, com as participantes sendo classificadas nas categorias nunca usaram, usaram no passado ou usam atualmente. As mulheres que nunca haviam usado a THM constituíram a categoria de referência.

Para identificar o momento em que o uso da THM foi iniciado em relação à menopausa, foi criada uma variável subtraindo a idade em que ocorreu a menopausa da idade de início da THM. O tempo de menopausa no início do tratamento foi dicotomizado em <10 e ≥10 anos e o tempo de uso de THM, em <5 e ≥5 anos, de acordo com os consensos atuais sobre os riscos e benefícios da THM para a saúde.^3^

As mulheres que referiram uso atual da THM responderam perguntas sobre o nome genérico ou a marca do hormônio em uso. Com base nessas informações, foram criadas as variáveis “tipo de hormônio” e “via de administração”. O tipo de hormônio foi classificado como estrogênio + progestágeno; estrogênio; progestágeno; estrogênio + testosterona; tibolona, e outros. A variável “via de administração” foi dicotomizada em “oral” e “não oral”. Para garantir que apenas a THM sistêmica fosse incluída na análise, as participantes que relataram usar apenas formulações de THM vaginais foram excluídas do estudo.

### Pressão arterial e hipertensão

A pressão arterial foi aferida utilizando-se um monitor de pressão arterial Omron HEM 705CPINT seguindo um período de repouso de 5 minutos, com a participante sentada, com os pés apoiados no chão, após esvaziamento vesical. O manguito foi escolhido em função da circunferência do braço da participante, sendo o braço esquerdo selecionado para essa aferição. Foram obtidas três aferições em um ambiente tranquilo com condições de temperatura controladas (20 a 24°C) e em intervalos de um minuto.^15^ A média das duas últimas aferições foi usada para analisar os níveis de pressão arterial, apresentadas aqui como pressão arterial diastólica e sistólica.

Foram consideradas hipertensas as participantes que apresentaram pressão arterial sistólica média ≥140mmHg e/ou pressão arterial diastólica média ≥90mmHg, de acordo com as Diretrizes da Sociedade Brasileira e Européia de Cardiologia ^16 , 17^ ou que referiram uso de anti-hipertensivos nas últimas duas semanas.

### Covariáveis

As participantes que receberam um diagnóstico de diabetes, ou que estavam em tratamento com insulina ou medicamentos hipoglicemiantes orais, foram classificadas como portadoras de diabetes. Além disso, um diagnóstico de diabetes foi feito na presença de níveis de glicose em jejum ≥126 mg/dl, teste de tolerância a glicose após 2 horas ≥200 mg/dl ou hemoglobina glicada ≥6,5%.^18^

As amostras foram coletadas para testes laboratoriais após jejum noturno de 12 horas. O teste de tolerância a glicose oral foi realizado administrando-se 75 gramas de solução de dextrosol. A glicose foi medida pelo método de hexoquinase, usando o sistema ADVIA Chemistry^®^, e a hemoglobina glicada foi medida por cromatografia líquida de alta pressão. Os níveis de colesterol lipoproteína de baixa densidade (HDL) e de triglicérides foram determinados por um método colorimétrico enzimático usando o sistema ADVIA Chemistry^®^, enquanto os níveis de colesterol lipoproteína de alta densidade (LDL) foram estimados pela equação de Friedewald. O perfil lipídico foi classificado com base nos níveis desejados de colesterol HDL (>40 mg/dl) e triglicérides (<150 mg/dl), e o limite superior para o colesterol LDL (<130 md/dl).^19^

A atividade física foi avaliada a partir dos domínios de lazer e deslocamento do *International Physical Activity Questionnaire* , um instrumento que foi validado para uso com adultos brasileiros.^20^ As participantes foram classificadas como “ativas” (atividade física vigorosa >60 minutos/semana atividade física moderada ≥150 minutos/semana) ou “inativas” (atividade vigorosa <60 minutos/semana e outras atividades menos intensas <150 minutos/semana).^21^

Balanças Toledo® e estadiômetros Seca® foram usados para medir peso e altura respectivamente,^15^ com as participantes usando roupas padronizadas do estudo durante a medição. O índice de massa corporal (IMC) foi calculado usando-se a fórmula *peso/altura* ^2^ e classificado como abaixo do peso/eutrófica (IMC <25 kg/m^2^), sobrepeso (25-29,9 kg/m^2^) ou obesidade (≥30 kg/m^2^). O consumo de álcool foi classificado como excessivo (≥140 gramas de álcool/semana) ou não excessivo (<140 gramas de álcool/semana).^22^

A variável *idade* foi analisada como variável contínua e categórica. A variável raça/etnia foi obtida com a resposta à seguinte pergunta: *“O censo brasileiro (IBGE) usa as categorias ‘negro, pardo, branco, amarela, e indígena para classificar a cor da pele ou a etnia das pessoas. Se você tivesse que responder ao censo do IBGE hoje, como você se classificaria em relação a sua cor ou etnia?”* As participantes que se autoidentificaram como “indígenas” (n =21) ou “amarelas” (n = 72) foram excluídas da análise devido ao pequeno número de sujeitos.

### Análise de dados

As características da amostra foram descritas como frequências absolutas e proporções. Para as variáveis quantitativas, medianas e faixas interquartis foram usadas, já que os dados não apresentaram distribuição normal como indicado pelo teste de Shapiro-Wilk. O teste qui-quadrado de Pearson foi usado para avaliar a associação entre aspectos relacionados a saúde e variáveis sociodemográficas em função de nunca terem usado, terem usado no passado, estarem em uso atual da THM. O teste exato de Fisher foi usado para comparar o tipo de hormônio de acordo com a presença de hipertensão. A pressão sistólica e diastólica mediana foi comparada usando-se o teste Kruskal-Wallis seguido do teste post-hoc de Dunn.

A associação entre a variável independente (THM) e a variável dependente (hipertensão) foi testada usando-se regressão logística multivariada. A modificação de efeito foi analisada usando termos do produto; entretanto, nenhuma das covariáveis foi identificada como modificadora do efeito. Possíveis variáveis de confusão foram avaliadas comparando-se as odds ratio (OR ) da associação bruta com a OR após o ajuste para as possíveis variáveis de confusão *idade* e *IMC* , sendo o parâmetro a diferença de pelo menos 10% entre as associações. Apenas a variável IMC foi identificada como fator de confusão na análise. Entretanto, com base em literatura consagrada e em sua relevância clínica, decidiu-se também levar a idade em consideração. O nível de significância adotado foi de 5% e o software Stata 12 foi usado durante a análise estatística.

### Aspectos éticos

Os comitês de análise internos de todos os institutos envolvidos no ELSA-Brasil aprovaram o protocolo do estudo, assim como o Comitê Nacional de Ética em Pesquisa. Todos os participantes assinaram um termo de consentimento informado. Os participantes que tiveram alterações clínicas detectadas pelo estudo foram encaminhados aos serviços de saúde indicados.

## Resultados

A idade mediana das 2.138 mulheres que participaram do estudo foi de 57 anos (FIQ 53-62). De acordo com os relatos próprios, 1.492 (69,8%) nunca usaram a THM, enquanto 457 (21,4%) usaram no passado e 189 (8,8%) referiram uso atual.

O uso de THM foi mais comum em mulheres com IMC <25 kg/m^2^, níveis de triglicérides <150 mg/dl, em mulheres menos inativas fisicamente, não fumantes e não diabéticas. Dentre as mulheres que referiram uso passado, 59,7% tinham ≥60 anos de idade, enquanto 54,5% das que estavam em uso atual tinham entre 50 e 59 anos de idade ( Tabela 1 ).

**Tabela 1:** – Características sociodemográficas, estilo de vida e condição de saúde das mulheres que passaram por menopausa natural, de acordo com o uso de terapia hormonal da menopausa. ELSA-Brasil, 2008-2010

Características	Nunca usaram n (%)	Usaram no passado n (%)	Usam atualmente n (%)	p-valor
**Idade**				**0,000**
40-49 anos	140 (9,4)	16 (3,5)	20 (10,5)	
50-59 anos	900 (60,3)	168 (36,8)	103 (54,5)	
≥60 anos	452 (30,3)	273 (59,7)	66 (35,0)	
**Etnia/cor da pele**				**0,000**
Negras	272 (19,4)	54 (12,6)	17 (9,1)	
Pardas	384 (27,4)	114 (26,6)	45 (24,2)	
Brancas	747 (53,2)	260 (60,8)	124 (66,7)	
**Escolaridade**				**0,000**
Ensino médio	805 (54,0)	192 (42,0)	50 (26,5)	
Ensino superior	687 (46,0)	265 (58,0)	139 (73,5)	
* Consumo excessivo de álcool				**0,243**
Não	1,436 (96,5)	444 (97,4)	179 (94,7)	
Sim	52 (3,5)	12 (2,6)	10 (5,3)	
**Tabagismo**				**0,000**
Nunca fumou	821 (55,0)	289 (63,2)	106 (56,1)	
Ex-fumante	427 (28,6)	120 (26,3)	68 (36,0)	
Fumante	244 (16,4)	48 (10,5)	15 (7,9)	
**Atividade física**				**0,001**
Inativas	1,204 (81,6)	337 (74,6)	138 (73,4)	
Ativas	271 (18,4)	115 (25,4)	50 (26,6)	
**Índice de massa corporal**				**0,000**
≤ 24,9 kg/m^2^	502 (33,6)	185 (40,5)	99 (52,4)	
25,0 - 29,9 kg/m^2^	517 (34,7)	183 (40,0)	71 (37,6)	
≥ 30,0 kg/m^2^	473 (31,7)	89 (19,5)	19 (10,0)	
**Diabetes**				**0,007**
Não	1,128 (75,7)	365 (79,9)	160 (84,7)	
Sim	363 (24,3)	92 (20,1)	29 (15,3)	
**Hipertensão arterial**				**0,000**
Não	880 (59,0)	260 (57,0)	139 (73,5)	
Sim	612 (41,0)	197 (43,0)	50 (26,5)	
**Doença cardiovascular**				**0,581**
Não	1,412 (94,8)	431 (94,3)	182 (96,3)	
Sim	78 (5,2)	26 (5,7)	7 (3,7)	
**Colesterol LDL**				**0,396**
<130 mg/dL	648 (43,4)	215 (47,0)	84 (44,4)	
≥130 mg/dL	844 (56,6)	242 (53,0)	105 (55,6)	
**Colesterol HDL**				**0,041**
>40 mg/dL	1,452 (97,3)	446 (97,6)	178 (94,2)	
≤40 mg/dL	40 (2,7)	11 (2,4)	11 (5,8)	
**Triglicérides**				**0,000**
<150 mg/dL	1,044 (70,0)	351 (76,8)	158 (84,0)	
≥150 mg/dL	448 (30,0)	106 (23,2)	31 (16,0)	

*Consumo excessivo de álcool: ≥140 gramas de álcool por semana; HDL: lipoproteína de alta densidade; LDL: lipoproteína de baixa densidade.

A prevalência da hipertensão era de 40,2%. Entre as mulheres hipertensas, 71,3% nunca haviam usado a THM, enquanto 5,8% estavam em uso atual. Entre as mulheres normotensas, 68,8% nunca haviam usado a THM, enquanto 10,9% referiram uso atual.

A tabela 2 mostra as associações brutas e ajustadas para idade e IMC entre o uso de THM e a presença de hipertensão. As mulheres que estavam em uso atual da THM apresentaram chances significativamente menores de ter hipertensão (OR=0,59; IC 95%: 0,41-0,85), em comparação com as que nunca usaram. Essa associação persistiu mesmo após ajustes adicionais para a via de administração (dados não apresentados nas tabelas).

**Tabela 2:** – Associação entre uso de terapia hormonal da menopausa e hipertensão ELSA-Brasil, 2008-2010

	Nunca usaram n=1492	Usaram no passado n=457	Usam atualmente n=189
**Hipertensão arterial n (%)**			
Não	880 (68,8)	260 (20,3)	139 (10,9)
Sim	612 (71,3)	197 (22,9)	50 (5,8)
**OR (IC 95%)**			
Bruto	1	1,08 (0,88-1,34)	0,51 (0,36-0,72)
Ajustado*	1	0,89 (0,71-1,13)	0,59 (0,41 – 0,85)

OR: Odds Ratio; *Ajustado para idade e índice de massa corporal.

**Figura 1:**
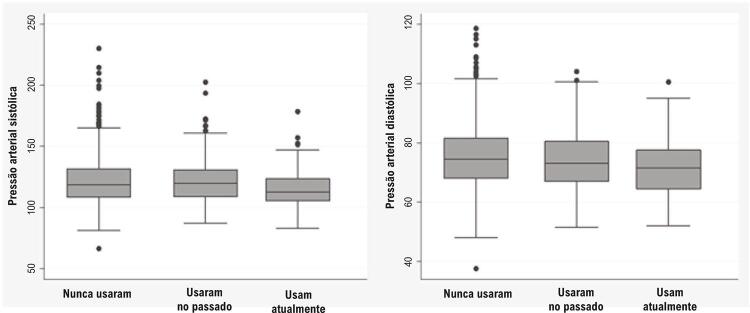
– Pressão arterial diastólica e sistólica mediana de acordo com o padrão de uso de terapia hormonal da menopausa.

Na análise comparativa dos níveis de pressão arterial sistólica de acordo com a exposição à THM, considerando mulheres hipertensas (usando anti-hipertensivos ou não) e normotensas, os resultados demonstraram que as mulheres em uso atual tinham a pressão arterial sistólica mediana mais baixa, em 113 mmHg, em comparação àquelas que nunca usaram, em 118,5 mmHg, e às que usaram no passado, em 120 mmHg (p=0,001). Além disso, o limite superior era notadamente mais baixo ( Figura 1 ). Foram encontradas diferenças estatisticamente significativas apenas entre as mulheres que nunca usaram/usam atualmente a THM (p=0,00) e entre as que usam atualmente/usaram no passado (p=0,00).

Entre as mulheres que estavam em uso atual e as que usaram anteriormente a THM, a maioria iniciou o tratamento com idade até 59 anos, com menos de 10 anos após a menopausa e a duração da terapia foi de até 5 anos, independentemente da hipertensão. Entretanto, a proporção de mulheres hipertensas foi maior entre as que começaram a THM após os 60 anos de idade e/ou 10 anos ou mais após a menopausa ( Tabela 3 ).

**Tabela 3:** – Idade no início da terapia hormonal da menopausa, tempo desde a menopausa e duração do uso da terapia hormonal da menopausa, de acordo com a presença de hipertensão entre mulheres em uso atual ou passado da terapia. ELSA-Brasil, 2008-2010

Características	Mulheres normotensas n (%)	Mulheres hipertensas n (%)	p-valor
**Idade no início da THM** ^**a**^			0,034
<60 anos	377(63,3)	219(36,7)	
≥60 anos	9(40,9)	13(59,1)	
**Tempo de menopausa no início da THM**			0,000
<10 anos	378 (63,7)	215 (36,3)	
≥10 anos	3 (17,6)	14 (82,4)	
**Duração do uso da THM**			0,927
<5 anos	252(62,4)	152(37,6)	
≥5 anos	142(62,0)	87(38,0)	

*^a^Terapia hormonal da menopausa. NB: Especificamente para essa análise, foram necessárias exclusões de algumas observações devido a dados faltantes e, por esse motivo, a soma pode variar para as diferentes variáveis.*

No grupo das mulheres em uso atual e que tinham hipertensão, o tipo de THM mais comum consistia em formulações que combinavam estrogênio-progestágeno seguidas de apenas estrogênio. Enquanto, em usuárias de THM normotensas, a tibolona também foi amplamente utilizada, bem como formulações que combinavam estrogênio-progestágeno. Todos os tipos diferentes de THM eram mais comuns em mulheres normotensas quando comparadas às mulheres hipertensas. Identificou-se que a maioria das mulheres (80,3%) usavam a administração por via oral. Entretanto, não houve associação estatisticamente significativa entre a via de administração e a presença de hipertensão ( Tabela 4 ).

**Tabela 4:** – Tipo de terapia hormonal da menopausa e a via de administração do regime atual, de acordo com a presença de hipertensão. ELSA-Brasil, 2008-2010

Características	Mulheres normotensas (n=138)	Mulheres hipertensas (n=50)	p-valor
	n (%)	n (%)	
**Tipo de hormônio**			0,024*
Estrogênio + progestágeno	65 (47,1)	18 (36,0)	
Estrogênios	28 (20,3)	16 (32,0)	
Progestágeno	3 (2,2)	-	
Estrogênio + testosterona	5 (3,6)	3 (6,0)	
Tibolona	35 (25,4)	8 (16,0)	
Outros	2 (1,4)	5 (10,0)	
**Via de administração**			0,190
Oral	114 (82,6)	37 (74,0)	
Não oral	24 (17,4)	(26,0)	

*Teste exato de Fisher. NB: Uma participante foi excluída devido a dados faltantes.

## Discussão

Os resultados indicam que o uso da THM não se relaciona à hipertensão arterial. As mulheres em uso atual da THM tiveram menores chances de ter hipertensão em comparação com as que usaram no passado e as que nunca usaram, independentemente de idade ou IMC. No entanto, esses achados devem ser analisados com cautela.

A possibilidade de que mulheres com problemas de saúde tenham tido menor probabilidade de serem prescritas com hormônios não pode ser descartada. As usuárias de THM tinham um perfil de saúde mais favorável, sendo mais saudáveis em quase todos os parâmetros avaliados aqui. Um estudo realizado no ELSA-Brasil demonstrou que mulheres com pelo menos uma contraindicação clínica à THM tinham probabilidade menor de serem expostas a esse tipo de medicamento.^23^ Portanto, a prescrição da THM pode ter sido mais restrita no caso de mulheres com hipertensão, uma vez que, embora a hipertensão em si não seja uma contraindicação formal, ela é frequentemente associada a doenças para as quais o uso de hormônio seria contraindicado. Não obstante, os presentes resultados estão alinhados com os achados do Estudo de Rancho Bernardo realizados na Califórnia com 1.044 mulheres, em que os níveis de pressão arterial das participantes que usavam THM no momento eram mais baixos do que os do grupo de controle.^24^

O estudo de Baltimore, com um tempo de acompanhamento de 10 anos, identificou que, embora os níveis de pressão arterial sistólica tenham aumentado em usuárias e não usuárias de THM, o aumento foi menos expressivo nas usuárias.^25^ No presente estudo, diferenças em níveis de pressão arterial medianos também foram encontrados entre usuárias e não usuárias de THM, especialmente em relação à pressão arterial sistólica, com uma diferença de 5,5 mmHg entre aquelas em uso atual e as que nunca usaram. Entretanto, em um ensaio clínico randomizado em que variações de pressão arterial foram determinadas por monitorização ambulatorial de pressão arterial (MAPA), foi identificada uma redução na pressão arterial diastólica e sistólica das usuárias de THM.^8^

Um estudo realizado na Finlândia avaliou o efeito de diferentes vias de administração da THM. Embora as vias oral e transdérmica propiciassem uma redução da pressão arterial sistólica diurna, essa redução foi mantida por mais tempo (6 meses) com a via oral, entretanto, tal estudo apenas analisou o efeito de curto prazo.^9^ Na presente amostra, apesar da maioria das usuárias de THM terem utilizado-a por via oral, não houve diferença significativa entre mulheres hipertensas e normotensas em função da via de administração.

Acredita-se que o estrogênio endógeno aja por um mecanismo fisiológico que pode promover uma redução da pressão arterial por meio de um efeito vasodilatador, como um aumento do óxido nítrico, inibição do sistema renina-angiotensina, redução da transcrição da enzima conversora da angiotensina, e regulação de vasoconstritores como a endotelina.^2 , 26^ Entretanto, apesar desse aparente benefício dos hormônios sexuais endógenos na saúde cardíaca das mulheres, estudos sobre o efeito do uso exógeno dessas substâncias nos níveis de pressão arterial geraram resultados conflitantes.

Um estudo transversal australiano que incluiu mulheres de 45 a 75 anos de idade identificou que o uso de hormônio estava associado a uma probabilidade significativamente maior de se ter hipertensão.^27^ Ademais, o ensaio clínico da Iniciativa de Saúde da Mulher ( *Women’s Health Initiative* - WHI), que avaliou mulheres entre 50 e 79 anos de idade, identificou que a THM levou a um pequeno aumento na pressão arterial sistólica em um período de acompanhamento de aproximadamente 5,2 anos.^10^ Em contrapartida, tanto o *Postmenopausal Estrogen/Progestin Intervention* ( *PEPI* ), que acompanhou mulheres entre 45 e 64 anos de idade durante um período de três anos,^28^ quanto um estudo realizado na Dinamarca^6^ com mulheres da mesma faixa etária, não identificaram nenhum efeito da THM na pressão arterial.

As diferenças encontradas nos estudos anteriores poderiam ser explicadas, primeiramente, pela diversidade das populações, cujas idades variavam de 45 a 79 anos. Segundo, os regimes, dosagens e formulações hormonais distintos, e os tempos de acompanhamento variaram entre 6 meses a 10 anos.^6 , 8 - 10^ Por último, as definições de hipertensão e métodos de aferição de pressão arterial diversificados, sendo usados PA ambulatorial,^10 , 25 , 28^ MAPA^8 , 9^ e autorrelato.^27^

Os ensaios clínicos que demonstraram uma associação entre THM e uma redução da pressão arterial ou um efeito neutro, possuíam amostras pequenas, compostas por mulheres mais jovens com período máximo de acompanhamento de um ano.^7 , 8^ Entretanto, os estudos em que se relatou um aumento da pressão arterial, tenderam a ter amostras maiores, com períodos mais longos de acompanhamento (até cinco anos), realizados com mulheres com idade mais avançada^10^ ou com doença arterial coronariana prévia.^11^

Além da associação encontrada no presente estudo entre THM e a menor ocorrência de hipertensão, é notável a baixa prevalência de THM. Apenas 8,8% das mulheres referiram uso atual, um achado que era esperado quando se considera que os dados desse estudo foram gerados após a publicação do Estudo do Coração e Reposição de Estrogênio/Progestina ( *Heart and Estrogen/Progestin Replacement Study* –HERS) e do estudo WHI. Essas publicações enfatizaram os riscos da THM e contribuíram para uma redução considerável em seu uso, com restrições para a prescrição de THM e a definição dos critérios para tratamento.^10 , 11^

O padrão de uso de THM visto aqui está alinhado com as recomendações atuais, já que a maioria das usuárias tinha menos de 60 anos de idade, quando a relação de risco-benefício da THM parece ser mais favorável, além de terem iniciado a THM com menos de 10 anos da menopausa e usaram o tratamento por períodos de até 5 anos.^3 , 4 , 29^ Nas mulheres que iniciaram o uso de hormônio mais tarde, a frequência de hipertensão encontrada foi maior; entretanto, essas mulheres constituíam uma minoria nessa amostra. O fato de que os limites de tempo recomendados estão sendo respeitados provavelmente garante mais proteção para as usuárias de THM.

As usuárias de THM no presente estudo tinham melhores condições de saúde, um estilo de vida mais saudável, e um grau de escolaridade elevado. Um estudo conduzido na Pennsylvania encontrou um perfil semelhante.^30^ Diante do padrão de indicadores de saúde entre as usuárias de THM, é preciso considerar a possibilidade de que a associação entre o uso do hormônio com a menor chance de hipertensão tenha sido influenciada pelo perfil de saúde dessas mulheres e não apenas pelo efeito da THM.

Apesar do benefício aparente da THM encontrado aqui, é importante enfatizar que, de acordo com recomendações atuais, a THM somente é indicada para o tratamento dos sintomas vasomotores da menopausa e não como uma estratégia para evitar doenças cardiovasculares e seus fatores de risco.^3 , 4^

Um dos pontos fortes do presente estudo é o tamanho substancial da amostra e o fato de que a amostra era composta de três grandes regiões geográficas do país. Entretanto, a generalização dos dados deve ser cautelosa, já que o ELSA-Brasil, apesar de sua amostra robusta e das semelhanças conhecidas entre os resultados deste estudo e os de pesquisas baseadas em população realizadas no Brasil, é composto por funcionários públicos que não representam o público em geral considerando suas características sociodemográficas.

Algumas limitações incluem a impossibilidade metodológica de se avaliar a causalidade reversa nas associações observadas aqui, bem como um possível viés de memória em relação aos dados relacionados a menopausa e ao início do uso da THM, que foram obtidos por questionários. Entretanto, qualquer viés que possa ter ocorrido seria mínimo, já que a menopausa é um evento marcante nas vidas das mulheres. Ademais, alguns fatores não avaliados, tais como o consumo de sódio, função renal e a dose usada nos regimes de hormônios, podem causar confundimento residual.

## Conclusão

Esses resultados sugerem que o uso atual da THM não está relacionado à hipertensão, especialmente em mulheres com um estilo de vida saudável e com menos de 60 anos de idade. Entretanto, estudos futuros podem esclarecer o efeito da THM na pressão arterial. A despeito das questões éticas que envolvem a realização de estudos pela delicada relação risco/ benefício da THM, estudos longitudinais podem ser mais apropriados para avaliar essa associação, além da possibilidade de se identificar efeitos de longo prazo após o término da terapia.
