# Supplementary material for: Terapia Hormonal e Hipertensão em Mulheres na Pós-Menopausa: Resultados do Estudo Longitudinal de Saúde do Adulto (ELSA-Brasil)
Source: Arq Bras Cardiol. 2022 May 4;118(5):905–13. [Article in Portuguese] doi: 10.36660/abc.20210218 (PMC9368886; doi:10.36660/abc.20210218)
Supplement: Translation

# Translation: Hormone therapy and Hypertension in Postmenopausal Women: Results from the Brazilian Longitudinal Study of Adult Health (ELSA-Brasil)

**Authors:** Luana Ferreira-Campos, Ligia Gabrielli, Maria da Conceição Chagas Almeida, Estela Maria Leão Aquino, Sheila Maria Alvim Matos, Rosane Harter Griep, Roque Aras

**Affiliations:** 1 Brazil Universidade Federal da Bahia - Programa de Pós-graduação em Medicina e Saúde, Salvador, BA – Brazil; 2 Brazil Universidade Federal da Bahia - Instituto de Saúde Coletiva, Salvador, BA – Brazil; 3 Brazil Fundação Oswaldo Cruz (Fiocruz) - Instituto Gonçalo Moniz, Salvador, BA – Brazil; 4 Brazil Fundação Oswaldo Cruz (Fiocruz) - Laboratório de Educação em Ambiente e Saúde, Rio de Janeiro, RJ – Brazil; 5 Brazil Universidade Federal da Bahia Faculdade de Medicina da Bahia, Salvador, BA – Brazil

**Keywords:** Postmenopause, Hormone Replacement Therapy

## Abstract

**Background:**

Hypertension is a major risk factor for cardiovascular morbidity and mortality in post-menopausal women. Although menopausal hormone therapy (MHT) is a very effective treatment for vasomotor symptoms during this period, the influence of this therapy on blood pressure is not yet clear.

**Objective:**

To evaluate the relationship between the use of MHT and hypertension in participants of the ELSA-Brasil.

**Methods:**

A cross-sectional study using the baseline ELSA-Brasil data in a cohort of 2,138 women who had experienced natural menopause. This study analyzed hypertension, defined as arterial pressure ≥140/90 mmHg or previous antihypertensive use, and use of MHT, with participants being classified into never, past, and current users. Associations were assessed using an adjusted logistic regression model, with statistical significance set at p<0.05.

**Results:**

Overall, 1,492 women (69.8%) had never used MHT, 457 (21.4%) had used it in the past, and 189 (8.8%) were current users. The use of MHT was more common in women who had a body mass index (BMI) <25 kg/m^2^and triglyceride levels <150 mg/dl, and who were physically less inactive, non-smokers, and non-diabetics. Current MHT users were less likely to have hypertension (OR=0.59; 95% CI: 0.41-0.85) compared to those who had never used MHT. In most cases, MHT was started at or before 59 years of age, within 10 years of becoming menopausal, and its use lasted for up to five years.

**Conclusion:**

Current MHT use was not related to hypertension, particularly in healthy women and in those under 60 years of age.

## Introduction

Hypertension is a major risk factor for cardiovascular morbidity and mortality in post-menopausal women.^1^ The menopausal transition accounts for changes that can increase the likelihood of hypertension and other cardiovascular risk factors. Indeed, changes in the endogenous sex hormones and in the physiology of aging itself may affect the cardiac function, cause arterial stiffness and insulin resistance, alter one’s lipid profile, and increase one’s bodyweight and central adiposity.^1 , 2^

Although menopausal hormone therapy (MHT) is the most effective treatment for vasomotor symptoms and for the genitourinary syndrome of menopause, and is a very effective treatment for the prevention of bone loss and fractures,^3^ other effects are also involved, and this treatment may be associated with cardiovascular risk markers.^4 , 5^ The risks and benefits of MHT use seem to depend on the type of hormone prescribed, the dose and the duration of use, the route of administration, and the moment at which treatment is begun.^3^

Findings regarding the effect of MHT on arterial blood pressure in women have been conflicting, with clinical trials reporting either a neutral effect^6 , 7^ or a protective effect with a reduction in blood pressure,^8 , 9^ while others with the same design have suggested a harmful effect with an increase in blood pressure levels.^10 , 11^

Because most of the studies dealing with this subject were performed with samples of North American and European women, there is a need to evaluate the effect of MHT on blood pressure in Brazilian women. Based on the hypothesis that MHT affects blood pressure levels, this study aimed to evaluate the relationship between MHT use and hypertension in women participating in the Brazilian Longitudinal Study of Adult Health (ELSA-Brasil).

## Materials and Methods

### Study design and population

This study analyzed baseline data from the ELSA-Brasil (2008-2010), a multicenter cohort that consisted of 15,105 civil servants working at public higher education and scientific research institutes in six Brazilian cities. Of these, 8,218 were women. Details on the study have already been published elsewhere.^12^

For the present analysis, 2,138 women who had experienced natural menopause and were normotensive, or who had received a diagnosis of hypertension after menopause, were evaluated. Conversely, a further 1,453 participants were excluded because they had undergone surgical or treatment-induced menopause, had a history of premature ovarian failure, had used MHT, or had received a diagnosis of hypertension prior to reaching menopausal age. In the analyses specifically related to the time of use or the date of starting MHT use, some participants were excluded due to missing data.

### Data collection

A trained team, certified to carry out each procedure, performed the data collection. A rigorous system of quality control was implemented.^13^ Face-to-face interviews were conducted using standardized questionnaires, and clinical and laboratory tests were conducted at the research centers.^12^

### Menopause and MHT

The participants who replied “no” to the following question: “Do you still menstruate?”, and those who also reported not having menstruated for over a year were considered menopausal.^14^ The type of menopause was investigated from the participant’s answer to the question: “Why do you no longer menstruate?” Age at menopause was determined from answers to the question: “How old were you when you menstruated for the last time?”

In relation to MHT use, the participants were asked: “Do you use or have you ever used drugs containing female hormones to relieve menopausal symptoms?” and “Are you currently using drugs containing female hormones to relieve menopausal symptoms?” These two questions were combined to obtain the exposure variable. The pattern of MHT use was evaluated categorically, with participants being classified into never, past, or current users, and the women who had never used MHT constituting the reference category.

To identify the time at which MHT use was begun in relation to he menopause, a variable was created by subtracting the age at which menopause occurred from the woman’s age upon beginning use of MHT. The time of menopause at the beginning of treatment was dichotomized into <10 and ≥10 years, and the time of MHT use into <5 and ≥5 years, in accordance with current consensuses on the risks and benefits of MHT to one’s health.^3^

Current MHT users were asked the generic or brand name of the hormone they were using. Based on this information, the variables “type of hormone” and “route of administration” were created. The type of hormone was classified as: estrogen + progestogen; estrogen; progestogen; estrogen + testosterone; tibolone; and others. The variable “route of administration” was dichotomized into “oral” and “non-oral”. To ensure that only systemic MHT was included in the analysis, participants reporting using only vaginal MHT formulations were excluded from the study.

### Arterial blood pressure and hypertension

Blood pressure was measured using an Omron HEM 705CPINT blood pressure monitor following a 5-minute resting period, with the participant seated, her feet resting on the ground, and after emptying her bladder. The cuff was chosen as a function of the participant’s arm circumference, with the left arm being selected for this measurement. Three measurements were taken in a quiet environment with controlled temperature conditions (20-24^o^C) and at intervals of one minute.^15^ The mean of the two last measurements was used for the analysis of blood pressure levels, presented here as systolic and diastolic blood pressure.

Participants whose mean systolic pressure was ≥140 mmHg and/or whose mean diastolic pressure was ≥90 mmHg, in accordance with the guidelines of the European and the Brazilian Societies of Cardiology,^16 , 17^ or who reported having used antihypertensive drugs in the preceding two weeks, were considered hypertensive.

### Co-variables

Participants who had received a diagnosis of diabetes or were under treatment with insulin or oral hypoglycemic drugs were defined as having diabetes. In addition, a diagnosis of diabetes was made in the presence of fasting glucose levels ≥126 mg/dl, or 2-hour levels in a glucose tolerance test≥200 mg/dl or a glycosylated hemoglobin ≥6.5%.^18^

Samples for laboratory tests were collected following 12-hour overnight fasting. The oral glucose tolerance test was performed by administering 75 grams of adextrose solution. Glucose was measured by the hexokinase method using the ADVIA Chemistry^®^system, and glycosylated hemoglobin was measured by high performance liquid chromatography. Triglyceride and high-density lipoprotein (HDL)-cholesterol levels were determined by an enzymatic colorimetric method using the ADVIA Chemistry^®^system, while low-density lipoprotein (LDL)-cholesterol levels were estimated by the Friedewald equation. The lipid profile was classified based on the desirable levels of HDL-cholesterol (>40 mg/dl) and triglycerides (<150 mg/dl), and the upper limit for LDL-cholesterol (<130 md/dl).^19^

Physical activity was evaluated from the leisure time and displacement domains of the International Physical Activity Questionnaire, an instrument that has been validated for use with adult Brazilians.^20^ The participants were classified as “active” (vigorous physical activity >60 minutes/week or moderate physical activity ≥150 minutes/week) or “inactive” (vigorous activity <60 minutes/week and other less intense activities <150 minutes/week).^21^

Toledo^®^scales and Seca^®^stadiometers were used to measure weight and height respectively,^15^ with the participants using standardized study clothing during measurements. Body mass index (BMI) as calculated using the formula *weight/height* ^2^and classified as underweight/normal weight (BMI <25 kg/m^2^), overweight (25-29.9 kg/m^2^), or obesity (≥30 kg/m^2^). Alcohol consumption was classified as excessive (≥140 grams of alcohol/week) or not excessive (<140 grams of alcohol/week).^22^

The variable *age* was analyzed as a continuous and categorical variable. The variable race/ethnicity was obtained by asking the following question: “ *The Brazilian census (IBGE) uses the categories ‘black, brown, white, Asian, or indigenous’ to classify a person’s color or ethnicity. If you had to answer the Brazilian census today, how would you describe your color or ethnicity?* ” Participants who self-identified as “indigenous” (n =21) or “Asian” (n = 72) were excluded from the analysis due to the low number of subjects.

### Data analysis

The characteristics of the sample are described as absolute frequencies and proportions. For the quantitative variables, medians and interquartile ranges were used, since the distribution of the data was not normal, as indicated by the Shapiro-Wilk test of normality. Pearson’s chi-square test was used to evaluate the association between health-related aspects and sociodemographic variables as a function of being a never, past, or current MHT user. Fisher’s exact test was used to compare the type of hormone according to the presence of hypertension. Median systolic and diastolic pressure was compared using the Kruskal-Wallis test, followed by Dunn’s post hoc test.

The association between the independent variable (MHT) and the dependent variable (hypertension) was tested using multivariate logistic regression. Effect modification was analyzed using product terms; however, none of the co-variables was found to be an effect modifier. Potential confounding variables were evaluated by comparing the odds ratios (OR) of the crude association with the OR, following adjustment for the possible confounding variables of *age* and *BMI* , with the parameter being a difference of at least 10% between the associations. Only the variable BMI was identified as a confounding factor in the analysis; however, based on the established literature and on its clinical relevance, it was decided to also take age into consideration. The significance level adopted was 5% and the Stata 12 software program was used throughout the statistical analysis.

### Ethical aspects

The internal review boards of all the institutes involved in the ELSA-Brasil approved the study protocol, as did the National Committee for Ethics in Research. All the participants signed an informed consent form. Participants who had clinical alterations detected by the study were referred to the referral health services.

## Results

The median age of the 2,138 women participating in the study was 57 years (IQR 53-62). According to self-reports, 1,492 (69.8%) were never users of MHT, while 457 (21.4%) were past users and 189 (8.8%) were current users.

MHT use was more common in women with a BMI <25 kg/m^2^and triglyceride levels <150 mg/dl, and in less physically inactive women, non-smokers and non-diabetics. Of the past users, 59.7% were ≥60 years of age, while 54.5% of the current users were 50-59 years of age ( Table 1 ).

**Table 1:** – Sociodemographic characteristics, lifestyle, and health status of the women who experienced natural menopause, according to the use of menopausal hormone therapy. ELSA-Brasil, 2008-2010

Characteristics	Never Users n (%)	Past Users n (%)	Current Users n (%)	p-value
**Age**				**0.000**
40-49 years	140 (9.4)	16 (3.5)	20 (10.5)	
50-59 years	900 (60.3)	168 (36.8)	103 (54.5)	
≥60 years	452 (30.3)	273 (59.7)	66 (35.0)	
**Ethnicity/skin color**				**0.000**
Black	272 (19.4)	54 (12.6)	17 (9.1)	
Brown	384 (27.4)	114 (26.6)	45 (24.2)	
White	747 (53.2)	260 (60.8)	124 (66.7)	
**Schooling**				**0.000**
High school	805 (54.0)	192 (42.0)	50 (26.5)	
University degree	687 (46.0)	265 (58.0)	139 (73.5)	
* Excessive alcohol consumption				**0.243**
No	1.436 (96.5)	444 (97.4)	179 (94.7)	
Yes	52 (3.5)	12 (2.6)	10 (5.3)	
**Smoking**				**0.000**
Never smoked	821 (55.0)	289 (63.2)	106 (56.1)	
Former smoker	427 (28.6)	120 (26.3)	68 (36.0)	
Smoker	244 (16.4)	48 (10.5)	15 (7.9)	
**Physical activity**				**0.001**
Inactive	1.204 (81.6)	337 (74.6)	138 (73.4)	
Active	271 (18.4)	115 (25.4)	50 (26.6)	
**Body mass index**				**0.000**
≤ 24.9 kg/m^2^	502 (33.6)	185 (40.5)	99 (52.4)	
25.0 - 29.9 kg/m^2^	517 (34.7)	183 (40.0)	71 (37.6)	
≥ 30.0 kg/m^2^	473 (31.7)	89 (19.5)	19 (10.0)	
**Diabetes**				**0.007**
No	1.128 (75.7)	365 (79.9)	160 (84.7)	
Yes	363 (24.3)	92 (20.1)	29 (15.3)	
**Arterial hypertension**				**0.000**
No	880 (59.0)	260 (57.0)	139 (73.5)	
Yes	612 (41.0)	197 (43.0)	50 (26.5)	
**Cardiovascular disease**				**0.581**
No	1.412 (94.8)	431 (94.3)	182 (96.3)	
Yes	78 (5.2)	26 (5.7)	7 (3.7)	
**LDL-cholesterol**				**0.396**
<130 mg/dL	648 (43.4)	215 (47.0)	84 (44.4)	
≥130 mg/dL	844 (56.6)	242 (53.0)	105 (55.6)	
**HDL–cholesterol**				**0.041**
>40 mg/dL	1.452 (97.3)	446 (97.6)	178 (94.2)	
≤40 mg/dL	40 (2.7)	11 (2.4)	11 (5.8)	
**Triglycerides**				**0.000**
<150 mg/dL	1.044 (70.0)	351 (76.8)	158 (84.0)	
≥150 mg/dL	448 (30.0)	106 (23.2)	31 (16.0)	

* Excessive alcohol consumption: ≥140grams of alcohol/week; HDL: high- density lipoprotein; LDL: low-density lipoprotein.

The prevalence of hypertension was 40.2%. Of the hypertensive women, 71.3% had never used MHT, while 5.8% were current MHT users. Of the normotensive women, 68.8% had never used MHT, while 10.9% were current users.

Table 2 shows the crude and the age- and BMI-adjusted associations between MHT use and the presence of hypertension. Current MHT users were significantly less likely to have hypertension (OR=0.59; 95% CI: 0.41-0.85) when compared to never users. This adjusted inverse association persisted even after making further adjustment for the route of administration (data not presented in tables).

**Table 2:** – Association between the use of menopausal hormone therapy and hypertension. ELSA-Brasil, 2008-2010

	Never user n=1492	Past user n=457	Current user n=189
**Arterial hypertension n (%)**			
No	880 (68.8)	260 (20.3)	139 (10.9)
Yes	612 (71.3)	197 (22.9)	50 (5.8)
**OR (95%CI)**			
Crude	1	1.08 (0.88-1.34)	0.51 (0.36-0.72)
Adjusted*	1	0.89 (0.71-1.13)	0.59 (0.41- 0.85)

OR: Odds Ratio; *Adjusted for age and body mass index.

In the comparative analysis of blood pressure levels according to exposure to MHT, considering hypertensive (using or not antihypertensive) and normotensive, results showed that current MHT users had the lowest median systolic blood pressure of 113mmHg, as compared to never users at 118.5mmHg, and to past users at 120mmHg (p=0.001). Furthermore, the upper limit was notably lower ( Figure 1 ). Statistically significant differences were found only between never/current use (p=0.00) and between current use/past use (p=0.00).

**Figure 1:** – Median systolic and diastolic blood pressure according to the pattern of use of menopausal hormone therapy.

Of the current and past users of MHT, the majority had begun treatment at or before 59 years of age, within 10 years of experiencing menopause, and the duration of therapy was up to 5 years, regardless of hypertension. Nevertheless, the proportion of hypertensive women was greatest among those who began MHT after 60 years of age and/or 10 years or more after menopause ( Table 3 ).

**Table 3:** – Age at beginning of menopausal hormone therapy, time since menopause, and duration of menopausal hormone therapy use according to whether or not hypertension was present in past and urrent users. ELSA-Brasil, 2008-2010

Characteristics	Normotensive women n (%)	Hypertensive women n (%)	p-value
**Age at beginning of MHT^a^**			**0.034**
<60 years	377(63.3)	219(36.7)	
≥60 years	9(40.9)	13(59.1)	
**Duration of menopause at beginning of MHT**			**0.000**
<10 years	378 (63.7)	215 (36.3)	
≥10 years	3 (17.6)	14 (82.4)	
**Duration of MHT**			**0.927**
<5 years	252(62.4)	152(37.6)	
≥5 years	142(62.0)	87(38.0)	

*a Menopausal hormone therapy. NB: Specifically for this analysis, exclusions of some observations were necessary due to missing data and for this reason, the sum may vary for the different variables.*

In the group of current users who had hypertension, the most common MHT type consisted of combined estrogen-progestogen formulations followed by estrogen alone. However, in normotensive users of MHT, tibolone was also widely used, as well as the combined estrogen-progestogen formulations. All the different types of MHT were more common in the normotensive women compared to the hypertensive women. The majority of the women (80.3%) were found to use the oral route of administration; nevertheless, there was no statistically significant association between the route of administration and the presence of hypertension ( Table 4 ).

**Table 4:** – Type of menopausal hormone therapy and the route of administration of current regimen, according to the presence of hypertension. ELSA-Brasil, 2008-2010

Characteristics	Normotensive women (n=138)	Hypertensive women (n=50)	p-value
	n (%)	n (%)	
**Type of hormone**			0.024*
Estrogen + progestogen	65 (47.1)	18 (36.0)	
Estrogens	28 (20.3)	16 (32.0)	
Progestogens	3 (2.2)	-	
Estrogens + testosterone	5 (3.6)	3 (6.0)	
Tibolone	35 (25.4)	8 (16.0)	
Others	2 (1.4)	5 (10.0)	
**Route of administration**			0.190
Oral	114 (82.6)	37 (74.0)	
Non-oral	24 (17.4)	26.0)	

* Fisher’s exact test. NB: One participant was excluded due to missing data.

## Discussion

The results indicate that the use of MHT is not related to arterial hypertension. MHT users were less likely to have hypertension compared to past or never users, regardless of age or BMI. Nevertheless, these findings must be analyzed with caution.

The possibility of women with health problems being less likely to be prescribed hormones, cannot be ruled out. MHT users had a more favorable health profile, being healthier in almost all the parameters evaluated here. A study conducted within the ELSA-Brasil showed that women with at least one clinical contraindication to MHT were less likely to be exposed to this type of medication.^23^ Therefore, the prescription of MHT may have been more restricted in the case of women with hypertension, since, although hypertension alone is not a formal contraindication, it is frequently associated with diseases for which hormone use would be contraindicated. Nonetheless, the present results are in agreement with the findings of the Rancho Bernardo Study conducted in California with 1,044 women, in which the blood pressure levels of participants in current use of MHT were lower than those of a control group.^24^

The Baltimore study, with a 10-year follow-up time, found that although systolic blood pressure levels increased in both the users and non-users of MHT, the increase was less expressive in the users.^25^ In the present study, differences in median blood pressure levels were also found between users and non-users of MHT, particularly in relation to systolic blood pressure, with a difference of 5.5 mmHg between current and never users. However, in a randomized clinical trial in which variations in blood pressure were determined by ambulatory blood pressure monitoring (ABPM), a decrease was found both in systolic and diastolic blood pressure in MHT users.^8^

A study conducted in Finland evaluated the effect of the different routes of administration of MHT. Although both the oral and transdermal routes of administration resulted in a decrease in daytime systolic blood pressure, this reduction was maintained for longer (6 months) with the oral route. However, that study only analyzed the short-term effect.^9^ In the present sample, although most of the MHT users used the oral route of administration, no significant differences were found between the hypertensive and normotensive women as a function of the route of administration.

Endogenous estrogen is believed to act through a physiological mechanism that can promote a reduction in arterial blood pressure via a vasodilatory effect, such as an increase in nitric oxide, inhibition of the renin-angiotensin system, a reduction in the transcription of angiotensin-converting enzyme, and the regulation of vasoconstrictors, such as endothelin.^2 , 26^ Nevertheless, despite this apparent benefit of endogenous sex hormones on women’s cardiac health, studies on the effect of the exogenous use of these substances on blood pressure levels have generated conflicting results.

A cross-sectional Australian study that included women of 45-75 years of age found that hormone use was associated with a significantly greater likelihood of having hypertension.^27^ Furthermore, the Women’s Health Initiative (WHI) clinical trial, which evaluated women of 50-79 years of age, found that MHT led to a small increase in systolic blood pressure over a follow-up period of approximately 5.2 years.^10^ Conversely, neither the Postmenopausal Estrogen/Progestin Intervention (PEPI), which followed women of 45-64 years of age over a three-year period,^28^ nor a study conducted in Denmark^6^ with women of the same age group, found any effect of MHT on arterial blood pressure.

The differences found in the previous studies could be explained first by the variations in the populations, whose ages ranged from 45 to 79 years. Secondly, the regimens, dosages, and hormonal formulations differed, and follow-up times ranged from 6 months to 10 years.^6 , 8 - 10^ Finally, the definitions of hypertension and methods of blood pressure measurement varied, with home monitoring,^10 , 25 , 28^ ABPM,^8 , 9^ and self-reporting^27^ being used.

In those clinical trials that reported an association between MHT and a reduction in blood pressure or a neutral effect, sample sizes were small, the women enrolled were younger, and participants were followed up for a maximum period of one year.^7 , 8^ Conversely, in those in which an increase in blood pressure was reported, studies tended to have larger sample sizes, involve longer periods of follow-up (up to five years), and be conducted in older women^10^ or in women with prior coronary heart disease.^11^

In addition to the association found in the present study between MHT and a lesser likelihood of hypertension, the low prevalence of MHT is noteworthy. Only 8.8% of the women were current users, a finding that was expected when bearing in mind that the data from this study were generated some years after the publication of the Heart and Estrogen/Progestin Replacement Study (HERS) and the WHI study. Those publications emphasized the risks of MHT and contributed to a considerable reduction in its use, with restrictions for the prescription of MHT and the establishment of criteria for treatment.^10 , 11^

The pattern of MHT use seen here is in agreement with current recommendations, since most users were under 60 years of age, when the risk-benefit ratio of MHT appears to be more favorable, had initiated therapy within 10 years of the menopause, and had used the treatment for periods of up to 5 years.^3 , 4 , 29^ In the women who initiated hormone use later, the frequency of hypertension was found to be greater; however, those women constituted a minority in this sample. The fact that the recommended time limits are being respected probably offers some protection to MHT users.

The MHT users in the present study had better health conditions, a healthier lifestyle and a better education level. A similar profile was found in a study conducted in Pennsylvania.^30^ Bearing in mind the pattern of health indicators among the users of MHT, the possibility has to be taken into account that the association between hormone use and a lesser likelihood of hypertension could have been affected by the health profile of these women and not only by the effect of MHT.

Despite the apparent benefit of MHT found here, it is important to emphasize that in accordance with current recommendations, MHT is only indicated for the treatment of the vasomotor symptoms of menopause and not as a strategy to prevent cardiovascular diseases and their risk factors.^3 , 4^

One of the strengths of the present study is its substantial sample size and the fact that the sample consisted of women from three large geographic regions of the country. Nevertheless, caution is required when making generalizations, since the ELSA-Brasil, despite its robust sample and the known similarities between the results of this study and those of population-based surveys conducted in Brazil, consists of civil servants who are not representative of the general public insofar as their sociodemographic characteristics are concerned.

Other limitations include the methodological impossibility of evaluating reverse causality in the associations observed here, as well as a possible memory bias with respect to the data concerning menopause and the beginning of MHT use, which were obtained using questionnaires. Nevertheless, any bias that may have occurred would be minimal, since the menopause is an important event in women’s lives. In addition, some factors not evaluated, such as sodium intake, kidney function, and the dose used in the hormone regimens, could have led to residual confounding.

## Conclusion

These results suggest that current MHT use is not related to hypertension, particularly in women with a healthy lifestyle and those under 60 years of age; however, future studies may clarify the effect of MHT on arterial blood pressure. Despite the ethical issues surrounding studies on MHT due to the delicate risk/benefit balance, longitudinal studies may be more appropriate to evaluate this association and may also include the possibility of identifying long-term effects following the end of MHT use.
